# Epidemiological and Clinical Profile of Fixed Pigmented Erythema at the Departmental University Hospital Center Borgou/Alibori (Benin)

**DOI:** 10.1155/drp/9911682

**Published:** 2026-02-02

**Authors:** Fabrice Akpadjan, Laura Dotsop, Nadège Agbessi, Christiane Koudoukpo

**Affiliations:** ^1^ Service de Dermatologie – Vénérologie du Centre de Dépistage et de Traitement de l’Ulcère de Buruli d’Allada, Faculté des Sciences de la Santé de l’Université d’Abomey-Calavi, Cotonou, Benin; ^2^ Service de Dermatologie-Vénérologie du Centre Hospitalier Universitaire Département du Borgou-Alibori, Faculté de Médecine de l’Université de Parakou, Parakou, Benin

**Keywords:** FPE, Parakou (Benin), self-medication, toxidermia

## Abstract

**Introduction:**

Fixed pigmented erythema (FPE) is a common toxidermia characterized by the appearance of one or more annular, erythematous and hyperpigmented spots, following the systemic administration of a drug. The main aim of this study was to describe the epidemiological and clinical aspects of fixed pigmented erythema at the Departmental University Hospital Center Borgou/Alibori (DUHC‐B/A) from 2009 to 2022.

**Methods:**

This was a descriptive cross‐sectional study with retrospective data collection, based on the records of patients seen in the Dermatology‐Venerology Unit for FPE. Initially, all files bearing the diagnosis of toxidermia were identified; then, those with the diagnosis of FPE with usable data were retained. Data were entered using EpiData 3.1 and analyzed using EpiData Analysis.

**Results:**

Sixty‐four patients were enrolled during the study period. The prevalence of FPE was 0.73%, with a male predominance. The most common drug identified was cotrimoxazole, followed by paracetamol and quinine. Over half of the patients (52.9%) were self‐medicating.

**Conclusion:**

Although FPE occurs rarely, it remains the most frequent toxidermia at the DUHC‐B/A. It can be severe in its generalized bullous form. Avoiding the practice of self‐medication could help reduce its prevalence.

## 1. Introduction

Fixed pigmented erythema (FPE) is a common toxidermia characterized by the appearance of one or more annular, oval, erythematous and hyperpigmented spots, following the administration of a systemic drug [1, 2]. Upon re‐exposure to the concerned drug(s), lesions may reappear on the same and/or different sites. The lesions leave behind postinflammatory hyperpigmentation [3]. The rash appears a few days to 2 weeks after initial exposure to the causative agent and 30 min to 16 h after re‐exposure [3]. The drugs involved vary according to the common illnesses and prescription habits in each part of the world. The list is not exhaustive [4, 5]. Cases of FPE to plants, dietary supplements, food colorants, preservatives and tinctures have been reported [3–6].

The precise mechanisms by which FPE occurs are unknown, but a cell‐mediated process involving CD8 T lymphocytes has been incriminated [7]. A genetic predisposition has been described, involving the HLA‐B22 and Cw1 antigens [8]. Histopathological examination shows interface dermatosis, vacuolar change, keratinocyte necrosis and pigment incontinence in the dermis. Mild acanthosis, hyperkeratosis, spongiosis, dermal edema and the presence of eosinophils and rare neutrophils may also be present [1, 3].

The aim of this study was to describe the epidemiological and clinical aspects of FPE in the Dermatology‐Venerology Unit of the Departmental University Hospital Center Borgou/Alibori (DUHC‐B/A).

## 2. Patients and Methods

This was a descriptive cross‐sectional study with retrospective data collection by review of the files of all patients seen in the Dermatology‐Venerology Unit for FPE irrespective of age and gender. The study period was from January 2009 to June 2022. Initially, all files with the diagnosis of toxidermia were identified, followed by those with the diagnosis of FPE with exploitable data. The dependent variable was FPE, and the independent variables were epidemiological and clinical data. Data were recorded and analyzed using EpiData 3.1 and IBM SPSS Statistics 21 software, respectively. Patient data confidentiality was protected.

## 3. Ethics Statement

This work was conducted in accordance with current ethical standards. The research protocol was approved by the Local Ethics Committee for Biomedical Research of the University of Parakou (LECBR‐UP).

## 4. Results

### 4.1. Epidemiological Data

During the study period, 123 cases of toxidermia were recorded in the Dermatology‐Venerology Unit of DUHC‐B/A, out of a total of 8829 patients who consulted during that period. Of the 123 cases, 64 patients suffered from FPE, representing a frequency of 52.03% of all toxidermia, and of 0.73% of all consultations recorded in the Dermatology‐Venerology Unit of DUHC‐BA from January 2009 to June 2022. The mean age of patients was 32.34 ± 18.79 years, with extremes ranging from 1 to 83 years. Patients aged 21–35 were the most represented, with a proportion of 34.4%. The study population comprised 29 women (45.3%), giving a sex ratio (M/F) of 1.20. The most common level of education was university (29.7%), followed by primary education (18.8%), secondary education (15.6%) and the unschooled (10.9%).

Most of the patients were pupils, students and apprentices (34.4%), followed by the unemployed (21.9%) and civil servants (14.1%).

### 4.2. Clinical Data

Seventy percent of the patients had no previous medical history (Table 1).

**TABLE 1:** Distribution of patients with FPE by medical history (Dermatology‐Venerology Unit, DUHC‐B/A; 2009–2022).

	**Number (*n* = 64)**	**Percentage %**

None	45	70.3
Sinusitis	6	9.3
High blood pressure	5	7.8
Asthma	4	6.2
Diabetes	1	1.6
Physical disability	1	1.6
Allergic rhinitis	1	1.6
Syphilis	1	1.6
Total	64	100

The most frequently incriminated drug was cotrimoxazole (14.0%), followed by paracetamol (9.3%) and quinine (4.6%), as shown in Table 2.

**TABLE 2:** Distribution of patients with FPE by therapeutic class and most frequently incriminated drugs (Dermatology‐Venerology Unit, DUHC‐B/A; 2009–2022).

	**Number (*N* = 64)**	**Percentage %**

Antibiotics (*n* = 14; 21.9%)		
Cotrimoxazole	9	14.0
Amoxicillin	2	3.1
Ceftriaxone	1	1.6
Metronidazole	1	1.6
Clarithromycin	1	1.6
Analgesics–NSAIDs (*n* = 9; 14.0%)		
Paracetamol	6	9.3
Metamizole sodium	1	1.6
Diclofenac	1	1.6
Ibuprofen	1	1.6
Anti parasitic drugs (*n* = 7; 10.9%)		
Quinine	3	4.6
Sulfadoxine–pyrimethamine	2	3.1
Artemether–lumefantrine	1	1.6
Artesunate	1	1.6

Self‐medication was not specified for 30 patients. Of those for whom it was specified, 52.9% were self‐medicating.

The time interval between medication intake and the onset of symptoms was 1–7 days for 10.9% of the patients (Table 3).

**TABLE 3:** Distribution of patients with FPE according to time interval between drug intake and onset of symptoms (Dermatology‐Venerology Unit, DUHC‐B/A; 2009–2022).

	**Number**	**Percentage %**

Not specified	49	76.6
Upon 7 days	7	10.9
In the first 24 h	5	7.8
In the first 4 weeks	2	3.1
More than a month	1	1.6
Total	64	100.0

In this study, macules were the most common dermatological lesions (93.8%), followed by scales (26.6%) and erosions (18.8%) as shown in Table 4.

**TABLE 4:** Distribution of FPE patients by dermatological lesions (Dermatology‐Venerology Unit, DUHC‐B/A; 2009–2022).

	**Number (*n* = 64)**	**Percentage %**

Macules	60	93.8
Scales	17	26.6
Erosions	12	18.8
Crust	4	6.3
Blisters	3	4.7
Papules	2	3.1
Vesicles	1	1.6
Edema	1	1.6

The most common sites of lesions were the thighs in 51.6% of the patients, followed by the thorax (37.5%) and face (34.4%) as shown in Table 5.

**TABLE 5:** Distribution of FPE patients by lesion site (Dermatology‐Venerology Unit, DUHC‐B/A; 2009–2022).

	**Number (*n* = 64)**	**Percentage %**

Thighs	33	51.6
Thorax	24	37.5
Face	22	34.4
Legs	22	34.4
Arms	21	32.8
Fore arms	21	32.8
Back	20	31.3
Hands	19	29.7
Abdomen	16	25.0
Genitalia	15	23.4
Feet	14	21.9
Neck	10	15.6
Bottom	9	14.1
Palms	7	10.9
Eyebrows	6	9.4
Nasal alae	6	9.4
Scalp	6	9.4
Feet soles	3	4.7

The most common associated sign was pruritus in 62.5% of the patients (Table 6).

**TABLE 6:** Distribution of patients with FPE according to associated signs (Dermatology‐Venerology Unit, DUHC‐B/A; 2009–2022).

	**Number (*n* = 64)**	**Percentage %**

Pruritus	40	62.5
Pain	10	15.6
Fever	2	3.1
Burn sensation	1	1.6

**FIGURE 1:**
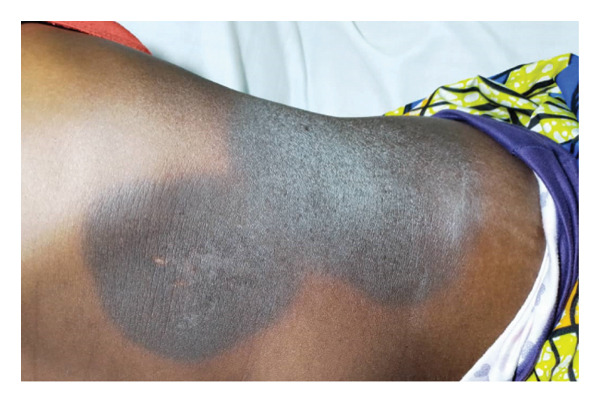
Fixed pigmented erythema in its typical form on the abdomen (source: *Dermatology-Venerology Unit DUHC-B/A*).

**FIGURE 2:**
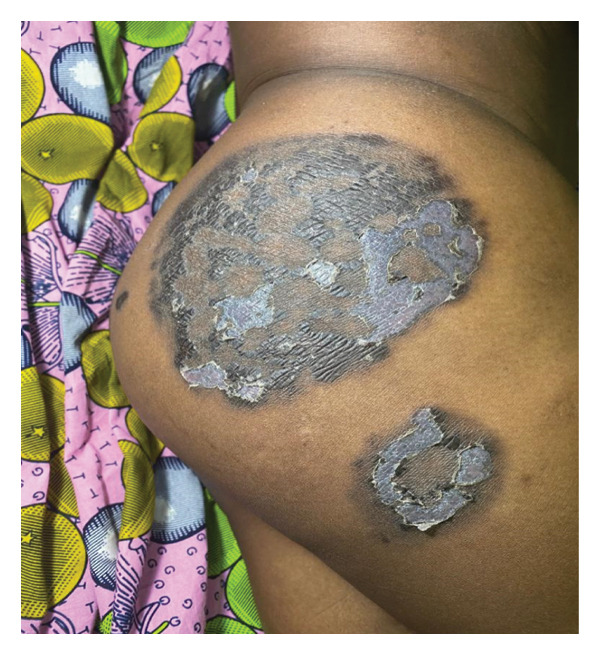
Bullous fixed pigmented erythema on the right buttock and thigh, scaling phase (source: *Dermatology-Venerology Unit DUHC-B/A*).

**FIGURE 3:**
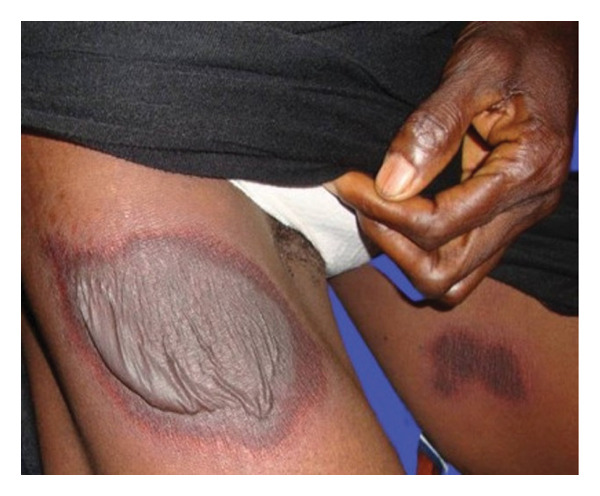
Bullous fixed pigmented erythema on the right thigh of a 65‐year‐old woman (source: *Dermatology-Venerology Unit DUHC-B/A*).

In this study, FPE presented in its typical form (Figure 1) in 52 patients (81.25%) and in its bullous form (Figures 2 and 3) in 12 patients (18.75%).

## 5. Discussion

The hospital frequency of FPE at DUHC‐B/A was 0.73% for all consultations recorded in the Dermatology‐Venerology Unit. This value is lower than those reported by Atadokpèdé et al. In Cotonou (Benin) in 2011 (1.1%) [9], Saka et al. in Togo in 2012 (1.1%) [10] and Ayanlowo in Nigeria in 2015 (1.8%) [11]. This low frequency could be explained by the fact that FPE is a benign toxidermia that does not worry patients in our setting to the point of motivating a consultation.

In this study, young people were the most affected, with a higher frequency in patients aged between 21 and 50 (34.4%). The mean age of patients was 32.34 ± 18.79 years. This is similar to the mean age reported by Atadokpèdé et al. (33 years), Ayanlowo et al. (32.12 ± 15.5 years) and Saka et al. (31.27 ± 14.01 years) [9–11]. The predominance of younger patients may be linked to an easy tendency to self‐medicate in this age group and to the repeated use of products known to cause toxidermia.

The study population was composed of 29 women (45.3%), giving a sex ratio (M/F) of 1.20. This male predominance was also found in the Togolese series [10]. On the other hand, a slight female predominance was reported in Cotonou (Benin) (M/F = 0.98) [9] and Nigeria [11]. Thus, the sex ratio varies between studies in the subregion. This difference may be linked to each population’s hospital‐attending habits and attitude to FPE.

In our series, 70% of the patients had no previous history of FPE. Among the remaining 30%, the most common antecedents were atopy and hypertension. In the study by Atadokpèdé et al., 50% of the patients had one or more pathological conditions associated with FPE. These were mainly atopy, polymedication, diabetes and HIV infection [9]. The role of pathological history in the occurrence of toxidermia in general, and EPF in particular, may be explained not only by changes in drug pharmacokinetics in certain pathological states, such as renal or hepatic insufficiency, but also by the frequency of polymedication in patients with an existing medical history [12]. As far as we know, there is no direct link between the onset of FPE and a history of atopy.

The most frequently incriminated drug was cotrimoxazole (14.0%), followed by paracetamol (9.3%) and quinine (4.6%). In African series, cotrimoxazole also accounts for a significant proportion of the drugs involved, ranging from 19.0% to 37.4% [9–11]. In European countries, on the other hand, anti‐infective drugs are not always the first responsible for FPE. Brahimi et al. in France [13] and Savin in the United Kingdom [14] found paracetamol to be the first drug responsible for FPEs, followed by nonsteroidal anti‐inflammatory drugs. The most incriminated drugs are also the most accessible in terms of cost and availability in health facilities, pharmacies and markets. These drugs in our countries are not yet subject to strict regulations governing their use.

Of the patients who provided information on the practice of self‐medication, 52.9% resorted to it. High percentages were also found by Atadokpèdé et al. and Saka et al. who reported 57% and 66.9%, respectively. These frequencies reflect the extent of this practice in our country, which can be explained by the poverty of the population, the sometimes limited access to healthcare, the phenomenon of street medicines available without prescription and illiteracy.

The time lapse between drug intake and the onset of FPE symptoms was 1–7 days in 10.9% of the patients. Atadokpèdé et al. and Saka et al. reported an average time lapse of 3 days (1–14 days) and 2.52 ± 3.04 days, respectively. From these results, we can see that FPE most often occurs less than a week after drug intake.

In this study, macules were the most common dermatological lesions (93.8%), followed by scales (26.6%) and erosions (18.8%). This high frequency of macules can be explained by the fact that macules are the most common lesion in FPE and are also the lesion found as a pigmentary sequela.

The most common sites were the thighs in 51.6% of the patients, followed by the thorax (37.5%) and face (34.4%). In the Nigerian and Togolese series, the most common sites were the trunk and lower limbs, the face and genitalia [10, 11].

In this study, FPE presented predominantly in its typical form (81.25%). This predominance of the hyperpigmented macular form was also found by Atadokpèdé et al. (83.87%) and Saka et al. (76.95%) [9, 10], in contrast to the French series by Brahimi et al. where bullous forms accounted for over 50% [13]. These same French authors also reported nonpigmented FPEs in 20.3% of the cases in their series [13].

Fixed drug eruption (FDE) is a T cell–mediated cutaneous adverse drug reaction characterized by the persistence of drug‐specific CD8^+^ resident memory T cells in previously affected skin sites, leading to lesion recurrence upon re‐exposure to the causative drug [15]. Individual susceptibility to FDE appears to be modulated by genetic factors, particularly polymorphisms in Class I human leukocyte antigen (HLA) molecules, which play a central role in antigen presentation to cytotoxic T lymphocytes [16, 17].

Associations between specific HLA alleles, mainly within the HLA‐B locus, and FDE induced by commonly implicated drugs such as antibiotics, nonsteroidal anti‐inflammatory drugs and paracetamol have been reported, although these associations are less consistent and less well established than those described for severe cutaneous adverse reactions, including Stevens–Johnson syndrome and toxic epidermal necrolysis [16, 18]. These genetic associations appear to be highly drug‐specific and population‐dependent, reflecting differences in HLA allele distribution across ancestral backgrounds.

The heterogeneous distribution of HLA alleles among populations may partly account for epidemiological differences in FDE incidence and in the spectrum of causative drugs reported worldwide [17]. Furthermore, immunogenetic variability may influence clinical phenotypes, including lesion extent, generalized or bullous forms and mucosal involvement [19]. In addition, postinflammatory hyperpigmentation, which is more pronounced in darker phototypes, may accentuate perceived phenotypic differences between populations, independently of the underlying immunological mechanisms [19].

At present, the lack of standardized genetic data precludes routine HLA screening in clinical practice for FDE. Multicenter studies involving diverse populations and detailed clinical phenotyping are needed to better define the immunogenetic determinants of this condition [17, 19].

## 6. Conclusion

This study confirms the relatively low frequency of FPE even though it is the most frequently observed toxidermia at the DUHC‐B/A and also the role of anti‐infective sulfonamides among FPE‐inducing drugs in sub‐Saharan Africa. It underlines the great responsibility of self‐medication in the occurrence of this toxidermia in Parakou. It is important to raise public awareness on the risks of self‐medication and to recognize the serious forms of FPE that can be life‐threatening. It should be noted that these results cannot be generalised to developed countries where practices are not the same.

## Funding

No funding was received for this manuscript.

## Conflicts of Interest

The authors declare no conflicts of interest.

## Data Availability

The data that support the findings of this study are available from the corresponding author upon reasonable request.
